# Reflectance Confocal Microscopy and Dermoscopy for the Diagnosis of Solitary Hypopigmented Pink Lesions: A Narrative Review

**DOI:** 10.3390/cancers16172972

**Published:** 2024-08-26

**Authors:** Luca Ambrosio, Anna Pogorzelska-Antkowiak, Chiara Retrosi, Giovanni Di Lella, Marco Spadafora, Iris Zalaudek, Caterina Longo, Giovanni Pellacani, Claudio Conforti

**Affiliations:** 1 Dermatology Unit, Department of Clinical Internal Anesthesiologic Cardiovascular Sciences, “Sapienza” University of Rome, 00185 Rome, Italy; pellacani.giovanni@gmail.com; 2 IDI-IRCCS, Dermatological Research Hospital, 00167 Rome, Italy; chiara.retrosi@gmail.com (C.R.); g.dilella@idi.it (G.D.L.); c.conforti@idi.it (C.C.); 3 EsteDerm Private Dermatology Clinic, 43-100 Tychy, Poland; annapogorzelska03@wp.pl; 4 Department of Dermatology, University of Modena and Reggio Emilia, 41121 Modena, Italy; spadafora.marco91@gmail.com (M.S.); longo.caterina@gmail.com (C.L.); 5 Skin Cancer Center, Azienda Unità Sanitaria Locale-IRCCS di Reggio Emilia, 42122 Reggio Emilia, Italy; 6 Department of Dermatology, Maggiore Hospital, University of Trieste, 34125 Trieste, Italy; iris.zalaudek@gmail.com

**Keywords:** pink lesions, reflectance confocal microscopy, dermoscopy, cherry angioma, Spitz nevus, clear cell acanthoma, dermatofibroma, basal cell carcinoma, actinic keratosis, amelanotic melanoma

## Abstract

**Simple Summary:**

The scarcity of specific clinical and dermoscopic criteria makes solitary pink skin lesions the most challenging to diagnose. Cherry angioma, clear cell acanthoma, dermal nevus, keloid, hypertrophic scar, and Spitz nevus are the most common benign lesions that share similar clinical and dermoscopic features. Furthermore, some malignant lesions, such as basal cell carcinoma, actinic keratosis, or amelanotic melanoma, can be indistinguishable from the above-mentioned benign pinkish lesions. Several studies have demonstrated the excellent diagnostic accuracy of reflectance confocal microscopy in increasing sensitivity and specificity compared to dermoscopy alone for the diagnosis of skin cancer. This study aimed to summarize the application of dermoscopy and RCM for the differential diagnosis of benign and malignant pinkish–reddish skin lesions, although, in suspicious lesions, the final diagnosis should always be confirmed after surgical excision and histopathological evaluation.

**Abstract:**

Diagnosing solitary pink skin lesions poses a significant challenge due to the scarcity of specific clinical and dermoscopic criteria. Several benign lesions, such as cherry angioma, clear cell acanthoma, dermal nevus, keloid, hypertrophic scar, and Spitz nevus, often exhibit similar clinical and dermoscopic features. This similarity extends to some malignant lesions, including basal cell carcinoma, actinic keratosis, and amelanotic melanoma, making differentiation difficult. Recent studies highlight the enhanced diagnostic accuracy of reflectance confocal microscopy (RCM), which offers increased sensitivity and specificity compared to dermoscopy alone for diagnosing skin cancer. This study aims to summarize the application of dermoscopy and RCM in distinguishing between benign and malignant pinkish–reddish skin lesions. The integration of RCM with traditional dermoscopic techniques improves the ability to accurately identify and differentiate these lesions. However, it is crucial to note that for any suspicious lesions, a final diagnosis must be confirmed through surgical excision and histopathological evaluation. This comprehensive approach ensures accurate diagnosis and appropriate treatment, highlighting the importance of combining advanced imaging techniques in clinical practice.

## 1. Introduction

The scarcity of specific clinical and dermoscopic criteria makes solitary pink skin lesions the most challenging ones to diagnose [1]. Many benign skin lesions, such as cherry angioma (CA), dermal nevus (DN), Spitz nevus (SN), clear cell acanthoma (CCA), and dermatofibroma (DF), present a reddish–pinkish color. However, even malignant ones, especially in the early stages and based on skin color phenotypes [2], may present as hypopigmented solitary lesions on clinical or dermoscopic examination, such as basal cell carcinoma (BCC), actinic keratosis (AK), and amelanotic melanoma (AM). Rare types of reactive lesions, such as spontaneous keloid (SK), may likewise show pink color in dermoscopy [3]. Reflectance confocal microscopy (RCM) is a noninvasive diagnostic technique that enables in vivo visualization of skin structures down to the papillary dermis with cellular-level resolution. Several studies have demonstrated its effectiveness in increasing sensitivity and specificity for diagnosing various types of skin lesions [4]. In this review, we aim to comprehensively summarize and evaluate the application of dermoscopy and RCM in the differential diagnosis of challenging pinkish–reddish skin lesions. By synthesizing existing research, this article seeks to provide a valuable resource for clinicians facing diagnostic uncertainty with these types of lesions.

## 2. Materials and Methods

A comprehensive literature search was conducted to identify relevant studies on the clinical, dermoscopic, and RCM criteria for diagnosing solitary pink skin lesions. The following databases were searched: PubMed, MEDLINE, Embase, and the Cochrane Library. The search was limited to articles published in English from January 2000 to December 2023. The search terms included “solitary pink skin lesions”, “cherry angioma”, “dermal nevus”, “Spitz nevus”, “clear cell acanthoma”, “dermatofibroma”, “basal cell carcinoma”, “actinic keratosis”, “amelanotic melanoma”, “spontaneous keloid”, “dermoscopy”, and “reflectance confocal microscopy”. The search specifically focused on articles describing hypopigmented (pink or red) lesions, both benign and malignant. Original articles, systematic reviews, meta-analyses, and clinical trials that looked into the RCM, dermoscopic, or clinical criteria of pink skin lesions were included in the review. If case reports, case series, or expert opinion articles offered comprehensive diagnostic criteria or novel insights, they were also taken into consideration. Articles were included if they investigated hypopigmented lesions, specifically focusing on the solitary pink skin lesions. The lesion had to be hypopigmented, with studies focused on lesions with other color characteristics excluded. Studies must have provided data on clinical, dermoscopic, or RCM criteria for differentiating between benign and malignant pink lesions. The literature search strategy was informed by the pre-determined list of diagnoses that were selected based on their clinical relevance and frequency in clinical practice, which informed the structure of the review and the categorization of the lesions into different subsections. Data were extracted from eligible studies on study characteristics, lesion features, and diagnostic methods and were qualitatively synthesized. The synthesis emphasized specific diagnostic criteria and assessed the efficacy of dermoscopy and RCM in improving diagnostic accuracy for solitary pink skin lesions. The RCM figures presented with each subsection were captured using the VivaScope^®^ 1500 or VivaScope^®^ 3000, while the dermoscopic images were obtained using the V-Track^®^ Vidix 4.0.

## 3. Results

### 3.1. Cherry Angioma

**Figure 1:**
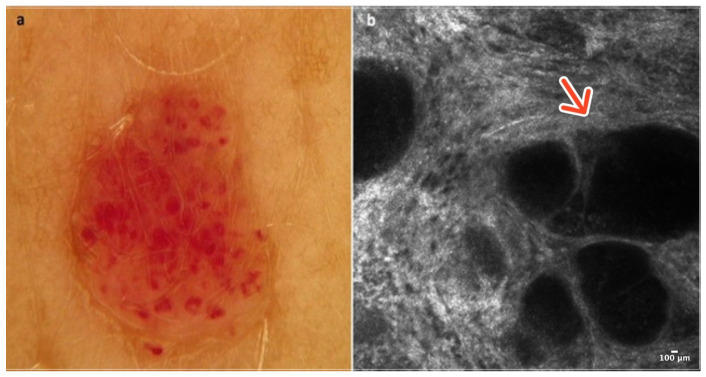
Cherry angioma. A 41-year-old woman presented with an erythematous papular lesion of the trunk. Dermoscopic image showing a well-demarcated pinkish lesion with white septa inside the lesion and red lacunes (**a**). RCM image with dark lacunes separated by bright septa (red arrow) (**b**). Courtesy, Anna Pogorzelska-Antkowiak, MD.

Cherry angioma is a benign, common skin tumor that develops from blood vessels. Dermoscopically well-demarcated red or pinkish lacunes separated by bright septa are observable (Figure 1a). Sometimes, there is a homogenous pinkish or reddish background with no other features. In most cases, CA is relatively easily recognizable on clinical examination, yet some of it can mimic other lesions, including melanoma [5]. 

In RCM, the epidermis reveals a typical honeycomb pattern. In the lower layers of the epidermis, the tops of dark lacunes are observable. In the dermoepidermal junction (DEJ) and the upper dermis, many dark lacunes separated by hyperreflective septa with blood cells flowing through them are common features [6] (Figure 1b); (Table 1).

### 3.2. Clear Cell Acanthoma

Clear cell acanthoma is an uncommon benign skin tumor, typically appearing as an asymptomatic, solitary, slowly enlarging pinkish-red papule or nodule on the lower limbs. The higher incidence of CCA in the lower extremities may suggest a reactive, inflammatory nature; however, its pathogenesis is still unknown. Histopathologically, they consist of clear epithelial cells containing glycogen [7].

**Figure 2:**
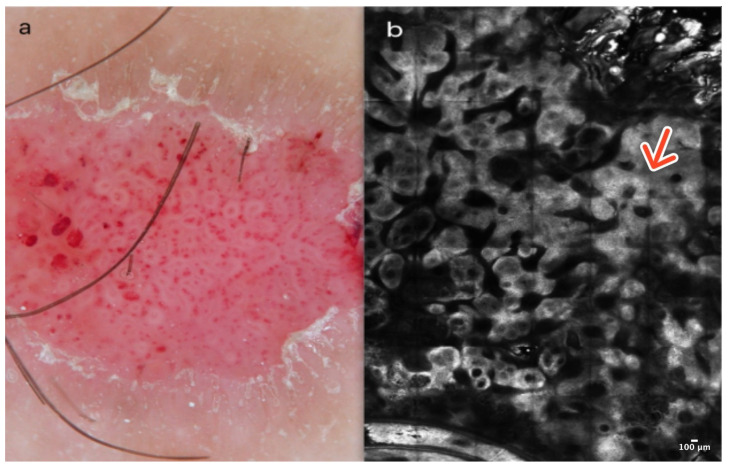
Clear cell acanthoma. A 38-year-old woman presented with an erythematous papular lesion on the left leg. Dotted vessels resembling a necklace and a scaly peripheral collarette in dermoscopy (**a**). At the level of the epidermis, the RCM image reveals acanthosis with papillomatosis (red arrow) in a well-defined lesion with a clear border (**b**). Courtesy, Prof. Caterina Longo.

Dermoscopically, CCA shows a typical vascular pattern: many dotted or glomerular vessels in a curvilinear and reticular distribution resembling a necklace [8] (Figure 2a). In RCM, CCA presents a well-defined lesion with a clear border, often demarcated by a collarette of hyperreflective hyperkeratotic cells. They exhibit features suggestive of psoriasis, including acanthosis with papillomatosis. More precisely, examination at the epidermal level reveals a loss of the honeycomb pattern alongside epidermal disarrangement. In addition, at the level of the dermoepidermal junction (DEJ) and the upper dermis, CCA exhibits numerous dilated blood vessels protruding into the epidermis [7,9] (Figure 2b); (Table 1).

### 3.3. Dermal Nevus

**Figure 3:**
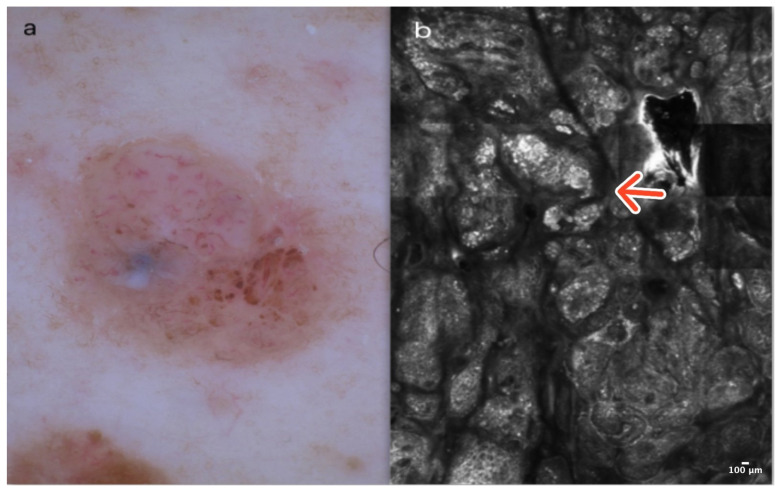
Dermal nevus. A 54-year-old male with a nodular lesion of the abdomen characterized by comma-like vessels and residual pigmentation on dermoscopy (**a**). RCM image showing clusters of homogenous, dense, and sparse nests at the DEJ (red arrow) (**b**). Courtesy, Prof. Caterina Longo.

Dermal nevus is a common, benign skin tumor localized primarily in the skin of the head, most commonly on the face. Clinically, it typically presents as a pinkish, soft, hairy papule or nodule, often reaching a diameter of a few millimeters. Dermoscopically, DN reveals comma-like vessels and pigment remnants with a cobblestone pattern [10] (Figure 3a).

In RCM, the epidermis shows a typical honeycomb pattern with dark hair follicles. DEJ reveals clusters of homogenous, dense, and sparse nests. At the level of the superficial dermis, corneal cysts consisting of very bright, amorphous material can be observed [11,12] (Figure 3b); (Table 1).

### 3.4. Dermatofibroma

**Figure 4:**
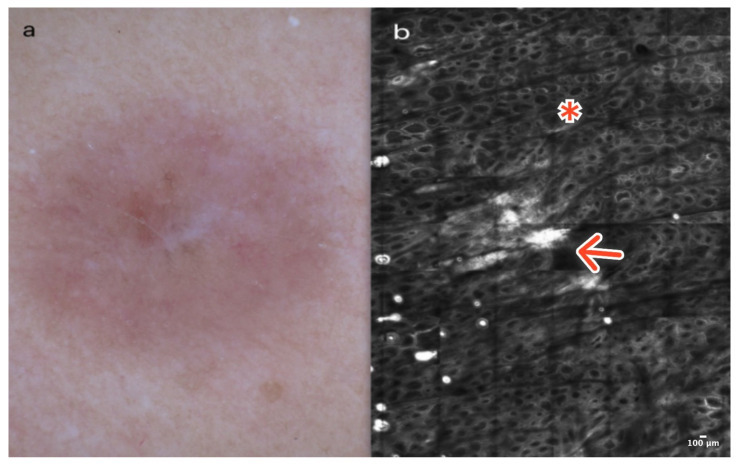
Dermatofibroma. A 36-year-old woman with a solitary, firm papule of the right deltoid. Small, central white scar-like area surrounded by a pinkish structureless zone in dermoscopy (**a**). RCM image showing thick reticulated collagen fibers in the central part of the lesion (red arrow) surrounded by edged and slightly bright papillae at the DEJ (red asterisk) (**b**). Courtesy, Prof. Caterina Longo.

Dermatofibroma is a common, benign skin lesion mostly located on the lower or upper limbs. The etiology of DF is still unknown. Clinically, DF presents as a pinkish or slightly pigmented, firm lesion that dimples on compression, usually measuring 3–5 mm in diameter. In dermoscopy, typical DF reveals a central whitish scar-like area surrounded by a pigmented network (Figure 4a). There are “atypical” pigmented forms of DF with globules or vessels, which can be diagnostically challenging [13].

In RCM, the epidermis of DF shows a honeycombed pattern and local “streaming” due to a scar-like area in the central part of the lesion [14,15,16]. At the DEJ, edged papillae and bright rings composed of monomorphic regular cells surrounding dark dermal papillae are observable. In the papillary dermis, the presence of thick, reticulated bundles of hyperreflective collagen fibers is characteristic. Dilated vessels are more frequent in cases of DF with a vascular pattern visible in dermoscopy as pinkish lesions [14,15,16] (Figure 4b); (Table 1).

### 3.5. Keloid and Hypertrophic Scar

**Figure 5:**
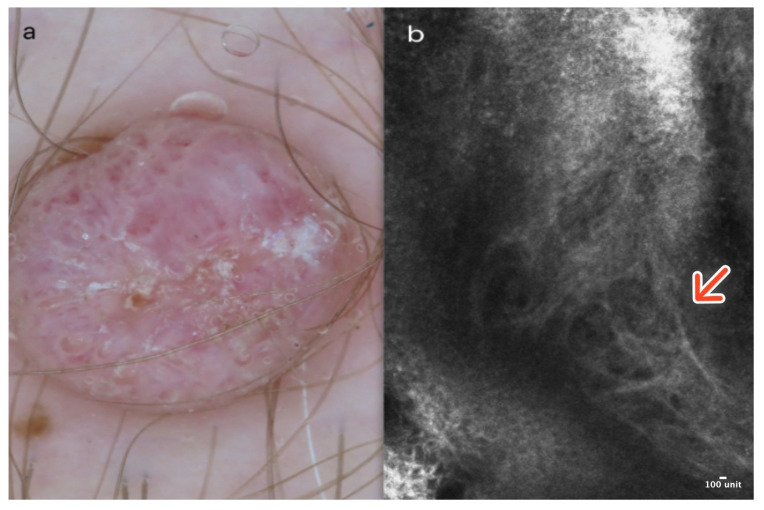
Keloid. A 43-year-old male presented with a firm, nodular lesion on the right thigh. Dermoscopic image showing irregular vessels on a homogenous pinkish background (**a**). RCM image reveals numerous coarse collagen fibers (red arrow) (**b**). Courtesy, Prof. Caterina Longo.

Keloid and hypertrophic scars are abnormal wound responses characterized by a chaotic fibroproliferative reaction that extends beyond or within the original wound margins. Occasionally, they can be linked with sensations of pruritus or pain and may resemble other pinkish–reddish skin lesions, such as amelanotic melanoma and basal cell carcinomas. Clinically, they usually present as dome-shaped, erythematous, and hard tumors. For these lesions, dermoscopy is somewhat nonspecific, and its usefulness lies more in the ability to rule out other conditions in the differential diagnosis. When assessed with a dermatoscope, arborizing, linear, or irregular vessels on a bright, homogenous background are observed [17] (Figure 5a).

In RCM, the epidermis displays a honeycombed pattern with local “streaming” and minor focal disarray. Within the dermoepidermal junction (DEJ) and the upper dermis, coarse or thick parallel collagen bundles, typically oriented predominantly perpendicular to the major axis of surgical scars, along with dilated vessels, are generally observable [18] (Figure 5b); (Table 1).

### 3.6. Spitz Nevus

**Figure 6:**
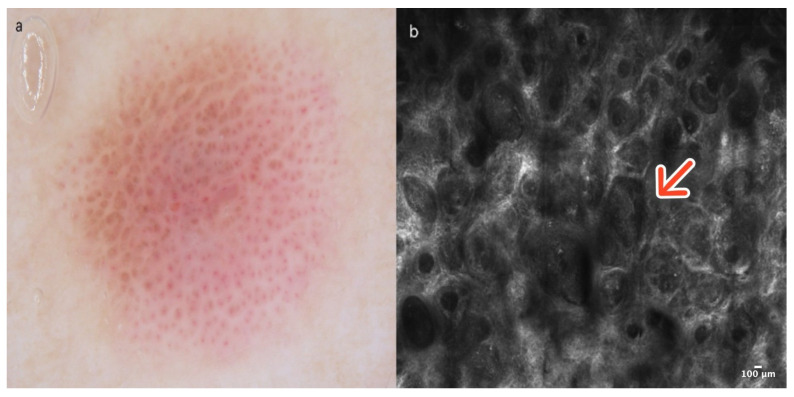
Spitz nevus. A 14-year-old boy presented with a pinkish macular lesion on the left arm. Dermoscopy is characterized by an inverse pigment network, dotted vessels, and pigment remnants (**a**). (**b**) In RCM, the DEJ reveals edged and non-edged papillae with spindle-atypical cells and homogeneous nests (red arrow). Courtesy, Anna Pogorzelska-Antkowiak, MD.

The Spitz nevi encompasses benign melanocytic tumors distinguished by a broad range of clinical, dermoscopic, and histopathologic features, whose main differential diagnosis is melanoma. They are typically described as pinkish-red papules that have recently appeared and grown rapidly, especially in very young individuals. There are four dermoscopic patterns of SN: starburst, globular with reticular depigmentation, dotted vessels with the inverse network in a macular lesion, and globular or coiled vessels with the inverse network in a nodular lesion [19]. In RCM, most SN presents an irregular honeycombed pattern, with the presence of a few roundish or dendritic atypical pagetoid cells generally located at the center of the lesion [20]. At the DEJ, edged and non-edged papillae with spindle-atypical cells are typical. Homogeneous, dense nests are commonly visible both at the DEJ and in the papillary dermis. Given that several features of SN are also detectable in melanomas, careful examination of all Spitzoid lesions is essential [21] (Figure 6b); (Table 1).

### 3.7. Basal Cell Carcinoma

Basal cell carcinoma is a common skin cancer in adult patients over 50 years of age.

**Figure 7:**
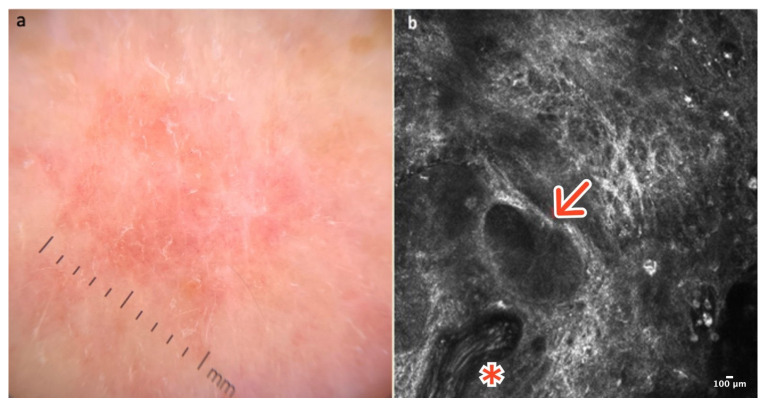
Basal cell carcinoma. A 65-year-old man presented with a pinkish macular lesion of the dorsum. Dermoscopy reveals a pinkish, structureless area with thin telangiectatic vessels (**a**). RCM image showing a thick, elongated vessel (red asterisk) and a dark tumor island (red arrow) with palisading of nuclei and peritumoral clefts (**b**). Courtesy, Anna Pogorzelska-Antkowiak, MD.

There are a few clinical types of BCC: nodular, morpheiform, superficial, and fibroepithelioma of Pinkus. BCC clinically, in most cases, shows itself as a pinkish–reddish nodule or papule. Dermoscopically pigmented BCC may reveal brown–gray globules and dots. Large blue–gray ovoid nests, spoke-wheel-like structures, or leaf-like pigmentation are helpful in the final diagnosis of pigmented BCC. Non-pigmented BCC may present only as a pinkish–reddish lesion with linear arborizing vessels, erosion, and ulceration [22] (Figure 7a).

RCM is very helpful in the diagnosis of BCC. Sensitivity and specificity for in vivo diagnosis of BCC are 82.9% and 95.7%, respectively [23]. Gonzalez and Tannous were the first to describe the features of BCC in reflectance confocal microscopy [24]. At the DEJ and in the papillary dermis, tumor islands are visible, comprising well-demarcated roundish dark or bright structures, which are separated from the surrounding dermis by slit-like spaces “clefting,” corresponding histopathologically to mucin deposition. Cells within the tumor island and sometimes in the overlying epidermis exhibit elongated nuclei oriented along the same axis, a phenomenon known as ‘streaming.’ Additionally, thickened and elongated blood vessels oriented parallel to the skin and in proximity to tumor islands are another typical feature [24,25,26]. Bright collagen bundles resulting from fibrosis and constituting the tumor stroma are frequently visible in the upper dermis [25,26] (Figure 7b); (Table 1).

### 3.8. Actinic Keratosis

**Figure 8:**
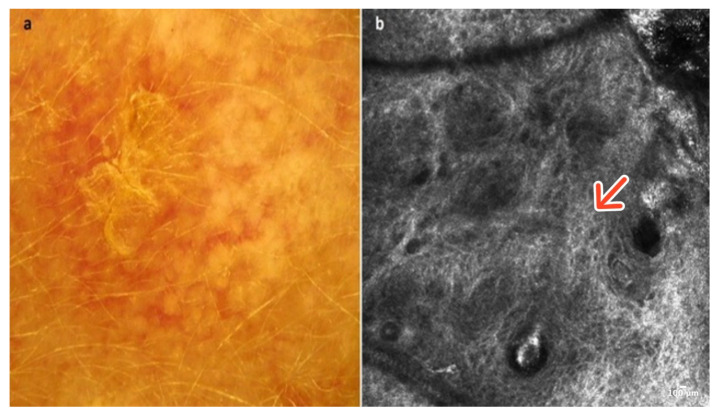
Actinic keratosis. A 72-year-old male presented with an erythematous–desquamative macular lesion of the frontal region. The dermoscopic image shows a red pseudonetwork or strawberry pattern with white–yellow scales (**a**). RCM image showing an irregular honeycombed pattern of the epidermis indicative of moderate-grade dyskeratosis (red arrow) (**b**). Courtesy, Anna Pogorzelska-Antkowiak, MD.

Actinic keratoses are keratinocyte neoplasms appearing on chronically UV-exposed skin, predominantly in individuals with a light phototype [27]. Clinically, they present as reddish-pink maculopapular lesions associated with variable degrees of scaling, sometimes appreciable only on palpation, and can be graded from mild to severe according to the 3-level Olsen scale. Dermoscopy of AK progresses from increased keratinizing features around follicles to pink or red “pseudonetwork” surrounding prominent hair follicles, also named the “strawberry” pattern. Grade III actinic keratosis shows poor dermoscopic features since the marked hyperkeratosis generally obscures the underlying lesion [28] (Figure 8a). RCM reveals hyperkeratosis with parakeratosis and increased thickness at the level of the stratum corneum, an irregular honeycomb pattern at the level of the epidermis, and dilated blood vessels within the dermal papillae [29] (Figure 8a). At the level of the papillary dermis, AK often shows inflammatory cells and collagen-thickened bundles with or without elastosis (Figure 8b); (Table 1).

### 3.9. Amelanotic Melanoma

**Figure 9:**
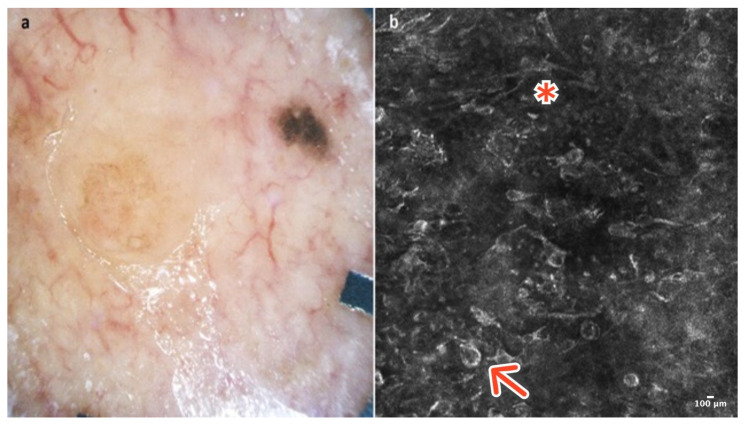
Amelanotic melanoma. A 53-year-old woman presented with a hypopigmented-pink macular lesion with minimal residual pigment of the upper trunk. Dermoscopy shows linear irregular vessels and remnants of pigmentation (**a**). RCM at the level of the epidermis reveals numerous roundish (red arrow) and dendritic (red asterisk) pagetoid cells within a disarranged epidermis (**b**). Courtesy, Anna Pogorzelska-Antkowiak, MD.

Amelanotic melanoma is not a very common type of melanoma, yet it is diagnostically challenging. Clinically, it presents as a reddish plaque or tumor located primarily on the skin exposed to UV radiation. In dermoscopy, AM typically exhibits dotted, linear, irregular, or polymorphic vessels evenly distributed on the surface of the lesion, often accompanied by an inverse network (Figure 9a). Some hypomelanotic melanomas may display a residual pigmented network. Therefore, when dealing with pinkish lesions, the presence of residual brownish pigmentation should be considered one of the first clues [30,31].

In RCM, most AMs demonstrate an atypical honeycomb and disarranged epidermal pattern, along with dendritic and hyporeflective atypical cells arranged in a pagetoid spread within the epidermis [31]. At the dermoepidermal junction, AM sometimes reveals non-edged papillae and the presence of atypical cells infiltrating the papillary dermis. There are numerous junctional nests and dermal nests, which can be dense and sparse and sometimes cerebriform [31,32,33] (Figure 9b); (Table 1).

## 4. Discussion

Clinical and dermoscopic evaluation currently serve as the primary diagnostic approach for skin lesions in dermatology. While highly effective for assessing pigmented lesions, this method often falls short for pinkish skin lesions, which lack specific diagnostic criteria, leading to a broader range of possible differential diagnoses. 

The introduction of noninvasive diagnostic techniques such as RCM, capable of visualizing in vivo structures at the epidermis, dermal–epidermal junction, and superficial dermis with cellular resolution on a horizontal plane, provides a unique opportunity to bridge the gap between dermoscopic patterns and histopathological observations. 

The combination of dermoscopy and confocal light microscopy is, therefore, essential for improving diagnostic accuracy and optimizing the clinical management of rosaceous skin lesions. This approach aims to maximize the surgical removal of malignant lesions while minimizing unnecessary interventions for benign lesions. Although aware of some inherent limitations of this methodological approach, such as the high cost of equipment, the expertise required to confidently execute this method, the time needed for image acquisition, and the persistence of some borderline cases difficult to diagnose even with this technique, our study summarizes some of the main indications and advantages of RCM in diagnosing pink skin lesions, often ambiguous in dermoscopy [34]. For the seven benign and three malignant pinkish skin lesions discussed in this paper, RCM generally provides significantly more information for diagnostic workup compared to what can be obtained solely from the analysis of vascular patterns or certain clues such as pigment remnants, erosions, ulcerations, and scales visualized in dermoscopy. When evaluating in detail the literature regarding the diagnostic accuracy of RCM for AK, published studies report sensitivities ranging from 91% to 100% and specificities from 78% to 100%. However, in most cases, these studies are characterized by a relatively small number of cases [35,36,37,38,39]. In contrast, the diagnostic accuracy of dermoscopy for AKs is associated with a sensitivity between 51.2% and 98.7% [40,41,42]. Another important factor to consider is the necessity of pretreating hyperkeratotic lesions in order to perform the technique and obtain diagnostically meaningful images. For BCC, it has been demonstrated that the presence of two or more of the major diagnostic criteria (tumor islands, palisading of nuclei, hyperreflective stroma, and peritumoral clefting) is 100% sensitive for BCC, while the simultaneous presence of four or more RCM criteria is associated with a specificity of 95.7% and sensitivity of 82.9% [23,43]. In contrast, according to a 2019 systematic review and meta-analysis, the sensitivity and specificity of dermoscopy for the diagnosis of BCC were 91.2% and 95%, respectively [44]. For AM, a systematic review and network meta-analysis showed that pooled sensitivity and specificity of dermoscopy for the diagnosis of AM are 61% and 90%, respectively, while for RCM, they are 67% and 89%, with a relative diagnostic odds ratio of RCM over dermoscopy of 4.69 [45].

## 5. Conclusions

Integrating reflectance confocal microscopy (RCM) with dermoscopy significantly improves the diagnostic accuracy of pinkish skin lesions, which often present challenges due to their nonspecific clinical and dermoscopic features. RCM enhances the ability to differentiate between benign and malignant lesions by providing detailed cellular-level imaging, complementing the broader pattern recognition of dermoscopy. RCM proves particularly valuable in identifying specific features of malignant tumors, such as basal cell carcinoma and amelanotic melanoma, that are difficult to detect with dermoscopy alone. This combined approach reduces unnecessary excisions of benign lesions and ensures accurate identification of malignancies, ultimately benefiting patient outcomes and healthcare efficiency. Incorporating RCM into routine practice enhances the precision of diagnoses, especially for challenging pinkish lesions.

## Figures and Tables

**Table 1:** Dermoscopic and RCM characteristics of pinkish skin lesions.

Lesion	Dermoscopic Characteristics	RCM Characteristics
Cherry Angioma	- Well-demarcated red or pinkish lacunes separated by bright septa. - Sometimes, a homogenous pinkish or reddish background with no other features.	- Epidermis with a typical honeycombed pattern. - Tops of dark lacunes are observable in the lower layers of the epidermis. - Dark lacunes separated by hyperreflective septa at the DEJ and upper dermis.
Clear Cell Acanthoma	- Vascular pattern with many dotted or glomerular vessels in a curvilinear and reticular distribution resembling a necklace.	- Well-defined lesion with a clear border and collarette of hyperreflective hyperkeratotic cells. - Loss of honeycomb pattern and epidermal disarrangement. - Dilated blood vessels protruding into the epidermis at DEJ and dermis.
Dermal Nevus	- Comma-like vessels. - Pigment remnants with a cobblestone pattern.	- Epidermis shows a typical honeycombed pattern with dark hair follicles. - DEJ reveals clusters of homogenous, dense, and sparse nests. - Superficial dermis shows corneal cysts with bright, amorphous material.
Dermatofibroma	- Central whitish scar-like area surrounded by a pigmented network. - Atypical pigmented forms may have globules or vessels.	- Epidermis shows a honeycombed pattern with local “streaming” due to a central scar-like area. - DEJ shows edged papillae and bright rings of monomorphic regular cells surrounding dark dermal papillae. - Papillary dermis shows thick reticulated bundles of hyperreflective collagen fibers and dilated vessels.
Keloid and Hypertrophic Scar	- Arborizing, linear, or irregular vessels on a bright, homogenous background.	- Epidermis shows a honeycombed pattern with local “streaming” and minor focal disarray. - DEJ and upper dermis show coarse or thick parallel collagen bundles, often oriented perpendicular to the major axis of surgical scars.
Spitz Nevus	- Four patterns: starburst, globular with reticular depigmentation, dotted vessels with the inverse network in macular lesions, and globular or coiled vessels with the inverse network in nodular lesions.	- Irregular honeycombed pattern with few roundish or dendritic atypical pagetoid cells. - DEJ shows edged and non-edged papillae with spindle-atypical cells. - Homogeneous dense nests visible at DEJ and in the papillary dermis.
Basal Cell Carcinoma	- Pigmented BCC: brown–gray globules, large blue–gray ovoid nests, spoke-wheel-like structures, or leaf-like pigmentation. - Non-pigmented BCC: pinkish–reddish lesion with linear arborizing vessels, erosion, and ulceration.	- DEJ and papillary dermis show tumor islands with well-demarcated roundish dark or bright structures separated by slit-like spaces (clefting). - Cells in tumor island may exhibit ‘streaming’. - Thickened and elongated blood vessels parallel to the skin. - Bright collagen bundles from fibrosis in the upper dermis.
Actinic Keratosis	- Strawberry pattern: pink or red “pseudonetwork” surrounding prominent hair follicles. - Grade III AK shows poor dermoscopic features due to marked hyperkeratosis.	- Hyperkeratosis with parakeratosis and increased stratum corneum thickness. - Irregular honeycomb pattern at the epidermal level. - Dilated blood vessels in the dermal papillae. - Inflammatory cells and thickened collagen bundles with or without elastosis in the papillary dermis.
Amelanotic Melanoma	- Dotted, linear, irregular, or polymorphic vessels evenly distributed on the lesion surface. - Inverse network. - Possible residual brownish pigmentation.	- Atypical honeycomb and disarranged epidermal pattern. - Presence of dendritic and hyporeflective atypical cells with a pagetoid spread within the epidermis. - DEJ shows non-edged papillae and atypical cells infiltrating the papillary dermis. - Junctional and dermal nests observable.

## Data Availability

The dataset is available on request from the authors.

## References

[1] Giacomel J., Zalaudek I. (2013). Pink Lesions. Dermatol. Clin..

[2] Zalaudek I., Argenziano G., Mordente I., Moscarella E., Corona R., Sera F., Blum A., Cabo H., Di Stefani A., Hofmann-Wellenhof R. (2007). Nevus Type in Dermoscopy Is Related to Skin Type in White Persons. Arch. Dermatol..

[3] Hofmann-Wellenhof R., Pellacani G., Malvehy J., Soyer H.P. (2012). Reflectance Confocal Microscopy for Skin Diseases.

[4] Haroon A., Shafi S., Rao B.K. (2017). Using Reflectance Confocal Microscopy in Skin Cancer Diagnosis. Dermatol. Clin..

[5] Piccolo V., Russo T., Moscarella E., Brancaccio G., Alfano R., Argenziano G. (2018). Dermatoscopy of Vascular Lesions. Dermatol. Clin..

[6] Gill M., González S. (2016). Enlightening the Pink: Use of Confocal Microscopy in Pink Lesions. Dermatol. Clin..

[7] Ardigo M., Buffon R.B., Scope A., Cota C., Buccini P., Berardesca E., Pellacani G., Marghoob A.A., Gill M. (2009). Comparing in vivo reflectance confocal microscopy, dermoscopy, and histology of clear-cell acanthoma. Dermatol. Surg..

[8] Cunha D.G., de Britto Pereira Kassuga-Roisman L.E., de Barros Silveira L.K.C., de Macedo F.C. (2018). Dermoscopic features of clear cell acanthoma. An. Bras. Dermatol..

[9] Venturi F., Dika E. (2024). Reflectance confocal microscopy of clear cell acanthoma: A novel insight to avoid invasive procedures. Ski. Res. Technol..

[10] Lu Q., Wang S., Wu T., Jiang G. (2021). Dermatoscopy and Reflective Confocal Microscopy for Facial Seborrheic Keratosis, Verruca Plana, and Nevus Pigmentosus. J. Coll. Physicians Surg. Pak..

[11] Guitera P., Pellacani G., Longo C., Seidenari S., Avramidis M., Menzies S.W. (2009). In Vivo Reflectance Confocal Microscopy Enhances Secondary Evaluation of Melanocytic Lesions. J. Investig. Dermatol..

[12] Braga J.C.T., Macedo M.P., Pinto C., Duprat J., Begnami M.D., Pellacani G., Rezze G.G. (2013). Learning reflectance confocal microscopy of melanocytic skin lesions through histopathologic transversal sections. PLoS ONE.

[13] Ferrari A., Argenziano G., Buccini P., Cota C., Sperduti I., De Simone P., Eibenschutz L., Silipo V., Zalaudek I., Catricalà C. (2013). Typical and atypical dermoscopic presentations of dermatofibroma. J. Eur. Acad. Dermatol. Venereol..

[14] Scope A., Ardigo M., Marghoob A.A. (2008). Correlation of Dermoscopic Globule-Like Structures of Dermatofibroma Using Reflectance Confocal Microscopy. Dermatology.

[15] Paolino G., Pampena R., Rizzo N., Di Nicola M.R., Mercuri S.R. (2022). Case Report of Dermoscopic Aspects and Reflectance Confocal Microscopy Description of Segmental Leiomyoma and Relative Management. Medicina.

[16] Konisky H., Sharma K., Suvarnakar A., Gregory N., Huho A. (2024). Deciphering dermatofibromas: A confocal and dermoscopic perspective for enhanced diagnostic precision. JAAD Case Rep..

[17] Yoo M.G., Kim I.-H. (2014). Keloids and Hypertrophic Scars: Characteristic Vascular Structures Visualized by Using Dermoscopy. Ann. Dermatol..

[18] Guida S., Pellacani G., Bencini P.L. (2019). Picosecond laser treatment of atrophic and hypertrophic surgical scars: In vivo monitoring of results by means of 3D imaging and reflectance confocal microscopy. Ski. Res. Technol..

[19] Lallas A., Apalla Z., Ioannides D., Lazaridou E., Kyrgidis A., Broganelli P., Alfano R., Zalaudek I., Argenziano G., the International Dermoscopy Society (2017). Update on dermoscopy of Spitz/Reed naevi and management guidelines by the International Dermoscopy Society. Br. J. Dermatol..

[20] Guida S., Pellacani G., Cesinaro A.M., Moscarella E., Argenziano G., Farnetani F., Bonamonte D., Longo C. (2016). Spitz naevi and melanomas with similar dermoscopic patterns: Can confocal microscopy differentiate?. Br. J. Dermatol..

[21] Pellacani G., Longo C., Ferrara G., Cesinaro A.M., Bassoli S., Guitera P., Menzies S.W., Seidenari S. (2009). Spitz nevi: In vivo confocal microscopic features, dermatoscopic aspects, histopathologic correlates, and diagnostic significance. J. Am. Acad. Dermatol..

[22] Wozniak-Rito A., Zalaudek I., Rudnicka L. (2018). Dermoscopy of basal cell carcinoma. Clin. Exp. Dermatol..

[23] Nori S., Rius-Díaz F., Cuevas J., Goldgeier M., Jaen P., Torres A., González S. (2004). Sensitivity and specificity of reflectance-mode confocal microscopy for in vivo diagnosis of basal cell carcinoma: A multicenter study. J. Am. Acad. Dermatol..

[24] González S., Tannous Z. (2002). Real-time, in vivo confocal reflectance microscopy of basal cell carcinoma. J. Am. Acad. Dermatol..

[25] Navarrete-Dechent C., DeRosa A.P., Longo C., Liopyris K., Oliviero M., Rabinovitz H., Marghoob A.A., Halpern A.C., Pellacani G., Scope A. (2019). Reflectance confocal microscopy terminology glossary for nonmelanocytic skin lesions: A systematic review. J. Am. Acad. Dermatol..

[26] Longo C., Guida S., Mirra M., Pampena R., Ciardo S., Bassoli S., Casari A., Rongioletti F., Spadafora M., Chester J. (2024). Dermatoscopy and reflectance confocal microscopy for basal cell carcinoma diagnosis and diagnosis prediction score: A prospective and multicenter study on 1005 lesions. J. Am. Acad. Dermatol..

[27] Rishpon A., Kim N., Scope A., Porges L., Oliviero M.C., Braun R.P., Marghoob A.A., Fox C.A., Rabinovitz H.S. (2009). Reflectance confocal microscopy criteria for squamous cell carcinomas and actinic keratoses. Arch. Dermatol..

[28] Moscarella E., Rabinovitz H., Zalaudek I., Piana S., Stanganelli I., Oliviero M.C., Lallas A., Ardigo M., Cota C., Catricalà C. (2015). Dermoscopy and reflectance confocal microscopy of pigmented actinic keratoses: A morphological study. J. Eur. Acad. Dermatol. Venereol..

[29] Tang Z., Kang L., Zhang Y., Huang J., Tong X., Zhou L., Zeng J. (2021). The diagnostic value of in vivo reflectance confocal microscopy in actinic keratosis. Ski. Res. Technol..

[30] Chi C.-C. (2020). Dermoscopy and reflectance confocal microscopy for early diagnosis of amelanotic/hypomelanotic melanoma: Still a long way to go?. Br. J. Dermatol..

[31] Spadafora M., Megna A., Lippolis N., Cavicchi M., Borsari S., Piana S., Guida S., Kaleci S., Chester J., Pellacani G. (2024). Dermoscopy and reflectance confocal microscopy of solitary flat pink lesions: A new combined score to diagnose amelanotic melanoma. J. Eur. Acad. Dermatol. Venereol..

[32] Losi A., Longo C., Cesinaro A., Benati E., Witkowski A., Guitera P., Pellacani G. (2014). Hyporeflective pagetoid cells: A new clue for amelanotic melanoma diagnosis by reflectance confocal microscopy. Br. J. Dermatol..

[33] Pizzichetta M.A., Canzonieri V., Rubegni P., Corneli P., Puglisi F., Zalaudek I., Cinotti E. (2021). Tips for difficult to diagnose hypomelanotic melanomas on reflectance confocal microscopy. J. Dermatol..

[34] Braga J.C.T., Scope A., Klaz I., Mecca P., González S., Rabinovitz H., Marghoob A.A. (2009). The significance of reflectance confocal microscopy in the assessment of solitary pink skin lesions. J. Am. Acad. Dermatol..

[35] Nguyen K.P., Peppelman M., Hoogedoorn L., Van Erp PE J., Gerritsen M.-J.P. (2016). The current role of in vivo reflectance confocal microscopy within the continuum of actinic keratosis and squamous cell carcinoma: A systematic review. Eur. J. Dermatol..

[36] Horn M., Gerger A., Ahlgrimm-Siess V., Weger W., Koller S., Kerl H., Samonigg H., Smolle J., Hofmann-Wellenhof R. (2008). Discrimination of actinic keratoses from normal skin with reflectance mode confocal microscopy. Dermatol. Surg..

[37] Incel P., Gurel M.S., Erdemir A.V. (2015). Vascular patterns of nonpigmented tumoral skin lesions: Confocal perspectives. Ski. Res. Technol..

[38] Ulrich M., Maltusch A., Rius-Diaz F., Roewert-Huber J., GonzALez S., Sterry W., Stockfleth E., Astner S. (2008). Clinical applicability of in vivo reflectance confocal microscopy for the diagnosis of actinic keratoses. Dermatol. Surg..

[39] Ulrich M., Maltusch A., Röwert-Huber J., González S., Sterry W., Stockfleth E., Astner S. (2007). Actinic keratoses: Non-invasive diagnosis for field cancerisation. Br. J. Dermatol..

[40] Zalaudek I., Giacomel J., Argenziano G., Hofmann-Wellenhof R., Micantonio T., Di Stefani A., Oliviero M., Rabinovitz H., Soyer H., Peris K. (2006). Dermoscopy of facial nonpigmented actinic keratosis. Br. J. Dermatol..

[41] Valdés-Morales K.L., Peralta-Pedrero M.L., Cruz F.J.-S., Morales-Sánchez M.A. (2020). Diagnostic Accuracy of Dermoscopy of Actinic Keratosis: A Systematic Review. Dermatol. Pract. Concept..

[42] Huerta-Brogeras M., Olmos O., Borbujo J., Hernández-Núñez A., Castaño E., Romero-Maté A., Martínez-Sánchez D., Martínez-Morán C. (2012). Validation of Dermoscopy as a Real-time Noninvasive Diagnostic Imaging Technique for Actinic Keratosis. Arch. Dermatol..

[43] Longo C., Borsari S., Pampena R., Benati E., Bombonato C., Raucci M., Mirra M., Di Stefani A., Peris K., Pellacani G. (2018). Basal cell carcinoma: The utility of in vivo and ex vivo confocal microscopy. J. Eur. Acad. Dermatol. Venereol..

[44] Reiter O., Mimouni I., Gdalevich M., Marghoob A.A., Levi A., Hodak E., Leshem Y.A. (2019). The diagnostic accuracy of dermoscopy for basal cell carcinoma: A systematic review and meta-analysis. J. Am. Acad. Dermatol..

[45] Lan J., Wen J., Cao S., Yin T., Jiang B., Lou Y., Zhu J., An X., Suo H., Li D. (2020). The diagnostic accuracy of dermoscopy and reflectance confocal microscopy for amelanotic/hypomelanotic melanoma: A systematic review and meta-analysis. Br. J. Dermatol..

